# *In vitro* fermentation of yeast cell walls (mannan-oligosaccharide) and purified β-glucans modulates the colonic microbiota of dogs with inflammatory bowel disease and demonstrates protective effects on barrier integrity and anti-inflammatory properties

**DOI:** 10.1371/journal.pone.0322877

**Published:** 2025-05-13

**Authors:** Jonas Ghyselinck, Lynn Verstrepen, Maike Rakebrandt, Sofie Marynissen, Sylvie Daminet, Massimo Marzorati

**Affiliations:** 1 ProDigest, Zwijnaarde, Belgium; 2 Leiber GmbH, Bramsche, Germany; 3 Small Animal Department, Faculty of Veterinary Medicine, Merelbeke, Belgium; 4 Center of Microbial Ecology and Technology (CMET), Ghent University, Ghent, Belgium; Washington State University - Spokane, UNITED STATES OF AMERICA

## Abstract

Inflammatory bowel disease (IBD) is characterized by a disruption of intestinal homeostasis, chronic inflammation, and dysbiosis. Prebiotic supplementation may be useful for managing IBD in dogs. The aim of the study is to investigate the effects of two prebiotics, Biolex MB40 or Leiber Beta-S, on the gut microbiota isolated from three dogs with IBD, using the Colon-on-a-plate technology. Biolex MB40 and Leiber Beta-S contain concentrated 1,3–1,6- β-D-glucan isolated from the *Saccharomyces cerevisiae* cell walls. Biolex MB40 also contains mannan-oligosaccharide (MOS). Wells of the Colon-on-a-plate set up were inoculated with fecal suspensions and supplemented with either Biolex MB40 and Leiber Beta-S, or no test product (blank). Following 48h incubation, bacterial metabolites were measured and 16S rRNA targeted gene sequencing was performed. Colonic supernatants were added to a Caco-2/THP1 co-culture model to evaluate their effects on barrier integrity upon inflammation-induced barrier disruption and interleukin (IL)-10 production. Acetate and propionate concentrations were significantly increased versus blank with Biolex MB40, and biologically relevant numerical increases were observed with Leiber Beta-S supplementation. A donor-dependent, biologically relevant increase in butyrate was observed with both test products versus blank. Alpha diversity and microbiota biomass were increased, as well as the abundance of the five predominant phyla with both test products relative to blank. The greatest increases in abundance were observed for the Bacteroidetes and Firmicutes phyla. Fermentation of both test products had a protective effect on the gut epithelial barrier (measured by transepithelial electrical resistance) that was donor dependent. IL-10 production was significantly increased with Biolex MB40 supplementation for all donors, and with Leiber Beta-S supplementation for one donor. These *in vitro* findings confirm a prebiotic effect for both products and suggest that supplementation with either Biolex MB40 or Leiber Beta-S may have beneficial effects on the gut microbiota of dogs with IBD.

## Introduction

Inflammatory bowel disease (IBD) in dogs is a collection of recurrent or chronic gastrointestinal disorders that lead to diarrhea, vomiting, and weight loss [1]. The pathogenesis of IBD is unknown, but features of the disease include the disruption of intestinal homeostasis, chronic inflammation, and an altered composition and function of the gut microbiota (i.e., dysbiosis) [2–8]. Gut microbiota dysbiosis is linked to chronic inflammation in dogs with IBD [6]. The canine gut microbiome is highly influenced by diet [9] and dietary interventions, such as a single protein diet, or inclusion of pre- and/or probiotics, can be useful for management of IBD in dogs [1].

Prebiotics are non-digestible fibers that are ‘selectively used by host microorganisms conferring a health benefit’ [10]. They are not digested or absorbed in the small intestine but are partially or fully fermented by bacteria in the colon [11,12]. Metabolites produced as by-products of colonic fermentation include the short-chain fatty acids (SCFAs) acetate, propionate, and butyrate, which are known for their beneficial effects on overall gut health [11–13]. SCFAs are able to reduce intestinal inflammation and improve the integrity of the intestinal barrier [13]. Studies have suggested that the prebiotic mannan-oligosaccharide (MOS) is associated with canine gut health and modulation of immune function [14–16]. The prebiotic effects of MOS are largely due to their ability to lower the abundance of pathogenic species, such as *Escherichia coli*, in the canine gut [14,16]. They also promote the growth of beneficial bacteria from the genera *Lactobacillus* [14] and *Bifidobacterium* [15,17].

The outer cell wall of *Saccharomyces cerevisiae* contains high levels of MOS and the inner layer is rich in *β*-glucan [18], which has also been shown to improve canine gut health. Particularly, *β*-glucans that have a linear backbone of *β*-1,3-glucosidic linkages substituted with a limited amount of *β*-1,6-linked side chains, which is typical of *β*-glucans derived from yeast, are known to have immunomodulatory activity [18–21]. Supplementation with β-1,3–1,6-D-glucan to dogs with IBD reduces Canine Inflammatory Bowel Disease Activity Index (CIBDAI) values, increases levels of the anti-inflammatory cytokine interleukin (IL)-10, and improves histopathological parameters [20].

Biolex MB40 (Leiber GmbH, Bramsche, Germany) is a commercially available yeast cell wall (*S. cerevisiae)*-prebiotic that consists of 1,3–1,6- β-D-glucan (25%) and MOS (20%). Leiber Beta-S (Leiber GmbH) is also commercially available and contains highly-purified 1,3–1,6- β-D-glucan (80%) isolated from the cell wall of *S. cerevisiae*. This *in vitro* study was conducted to investigate the impact of Biolex MB40 or Leiber Beta-S supplementation on the activity and composition of the gut microbiota of three dogs with IBD, and to assess the resulting impact of these changes on the host gut barrier integrity and immune response.

## Materials and methods

### Animal ethics statement

The authors confirm that the ethical policies of the journal, as noted on the journal’s author guidelines page, have been adhered to. Fecal samples were obtained during regular work-up for chronic diarrhea, therefore EC approval was not needed. Owners signed a written informed consent allowing usage of ‘left overs’ for research purposes. No ethical approval by an Institutional Animal Care and Use Committee was deemed necessary.

### Fecal samples

Fecal samples were collected from three canine (*Canis lupus familiaris*) donors with chronic gastrointestinal signs and histopathological findings suggestive for IBD. Clinical and histopathological data are described in S1 Table. Donors were one seven year-old Boxer, one two year-old crossbreed, and one two year-old Cavalier King Charles Spaniel. Fecal samples were collected immediately after defecation in airtight containers, in which an AnaeroGen^TM^ sachet (Thermo, Waltham, USA) was inserted to generate an anaerobic atmosphere. In the lab, the fecal samples were processed as 15% (w/v) fecal suspensions in anaerobic PBS buffer (8.8 g/L K2HPO4, 6.8 g/L KH2PO4, 0.1 g/L sodium thioglycolate and 15 mg/L sodium dithionite), and mixed in a 1:1 ratio with a modified version of the cryoprotectant developed by Hoefman et al. [22] to a final fecal concentration of 7.5% (w/v). The suspensions were then flash frozen and preserved at –80˚C until needed. Prior to initiating the experiment, an aliquot was defrosted and immediately added to the experimental wells.

### Colon-on-a-plate

The Colon-on-a-plate system mimics the well-established short-term batch fermentation model, but on a miniaturized scale. This system provides detailed mechanistic insights on how test products interact with the gut microbiota and allows for assessment of direct and/or indirect effects of the test product on host response. This miniaturized system uses deep well plates, making the volume per test condition 10-fold lower than traditional short-term batch fermentation assays. This allows testing of a greater number of treatments and testing of the fecal microbiome of multiple donors to account for interpersonal differences.

To initiate the experiment, inside the anaerobic workstation (DW Scientific, West Yorkshire, UK) wells of the Colon-on-a-plate (24-well plates with 10.4 ml volume; Thomson Instrument Company, Oceanside, California, USA) were filled with a nutritional medium (nutritional blend PD03; ProDigest, Ghent, Belgium) to represent the colon environment. Next, a single dose of Biolex MB40 or Leiber Beta-S (3.5 g/L; Leiber GmbH) was added to respective wells, corresponding to an *in vivo* dose of 56 mg/kg for either compound, assuming an average 10 kg body weight (medium sized dog). Finally, 10% (v/v) of cryopreserved fecal inoculum containing 7.5% (w/v) fecal material from a dog diagnosed with IBD was added to each well as a microbial source. All work was done in an anaerobic chamber with carefully monitored oxygen levels to guarantee that anaerobiosis was maintained. Additionally, each solution was boiled or sparged with nitrogen gas to drive out oxygen before introduction into the reactors. This study utilized fecal material from 3 canine donors. The total volume in each well was 9 mL. Plates were incubated in an anaerobic atmosphere at 39˚C for 48h. Each condition was tested in triplicate to account for technical variation (3 donors, 2 treatments, 1 blank; each in triplicate). Samples were collected 48h after the start of the experiment and assessed for concentrations of SCFAs (acetate, propionate, and butyrate) and lactate, community composition, and for use in co-culture experiments to study effects on transepithelial electrical resistance (TEER) and anti-inflammatory cytokine production.

### Assessment of microbial community activity

Quantitative analysis of the SCFA was done using capillary gas chromatography coupled with a flame ionization detector. The isolation of SCFAs was performed by liquid-liquid extraction using previously reported methods [23]. Lactate concentrations were determined using the Enzytec™ kit (R-Biopharm, Darmstadt, Germany). Each measurement was done in single repetition.

### Assessment of microbial community composition

Changes in microbial community composition were evaluated using 16S rRNA targeted gene sequencing (Illumina). Primers spanning two hypervariable regions (V3–V4) of the 16S rRNA gene were used (341F, 5′-CCTACGGGNGGCWGCAG-3′; 785R, 5′-GACTACHVGGGTATCTAAKCC-3′). A pair-end sequencing approach was used to sequence 2 × 250 bp, resulting in 424 bp amplicons, which are taxonomically more informative than smaller fragments. Read assembly and cleanup was largely derived from the MiSeq SOP described by the Schloss lab [24]. Briefly, mothur (v.1.44.3) was used to assemble reads into contigs, perform alignment-based quality filtering (alignment to the mothur-reconstructed SILVA SEED alignment, v138), remove chimeras (vsearch v2.13.3), assign taxonomy using a naïve Bayesian classifier and SILVA NR v138_1, and cluster contigs into Operational Taxonomic Units (OTUs) at 97% sequence similarity. All sequences that were classified as Eukaryota, Archaea, chloroplasts, or mitochondria, or that could not be classified, were removed. The most abundant sequence within an OTU was chosen as the representative. Reads with maximum abundances of five or less across samples were removed, as they are considered artefacts or bacteria with no biological impact.

Samples analyzed by 16S rRNA targeted gene sequencing were also analyzed using flow cytometry to determine the number of total bacterial cells. Samples were analyzed on a BD Accuri C6 Plus Flow Cytometer (BD Biosciences, Franklin Lakes, New Jersey, USA) using the high flow rate. Bacterial cells were separated from medium debris and signal noise by applying a threshold level of 700 on the SYTO channel. Appropriate parent and daughter gates were set to determine all populations. Relative abundances of each population in a sample were multiplied with the total cell count obtained by flow cytometry to convert proportional values obtained using 16S rRNA targeted gene sequencing to absolute quantities [25].

### Caco-2/THP1 co-culture model

Caco-2 cells (HTB-37; American Type Culture Collection, Manassas, Virginia, USA) were maintained in Dulbecco’s Modified Eagle Medium (DMEM; Merck Life Science B.V., Darmstadt, Germany) supplemented with 10 mM HEPES (Merck Life Science B.V.) and 20% (v/v) heat-inactivated fetal bovine serum (HI-FBS; Gibco, Life Technologies Europe B.V., Thermo Fisher Scientific, Sjælland, Denmark). THP1-Blue cells (InvivoGen, San Diego, California, USA) were cultured in Roswell Park Memorial Institute (RPMI) 1640 medium (Merck Life Science B.V.) supplemented with 10 mM HEPES, 1 mM sodium pyruvate (Merck Life Science B.V.), and 10% (v/v) HI-FBS. Cells were cultured at 37°C in a humidified atmosphere of air:CO_2_ (95:5, v/v).

The co-culture was performed using the method described by Daguet et al [26]. Caco-2 cells were cultured on 24-well inserts (0.4 µm, cellQART; Sabeu GmbH & Co. KG, Northeim, Germany) for 14 days to obtain a functional cell monolayer. THP1-Blue cells were differentiated in 24-well plates with 100 ng/mL phorbol 12-myristate 13-acetate (PMA; Merck Life Science B.V.) for 48h. In the next step, TEER of the Caco-2 monolayers was measured and the inserts were transferred to the activated THP1 cells. Sterile-filtered (0.22 µm) and 1:5 (v/v) diluted colonic suspensions were applied (apical) to the co-culture system. After 24h, TEER was measured again and 500 ng/mL lipopolysaccharide (LPS, E. coli K12; InvivoGen) was used to stimulate the basolateral THP1 cells for 6h. Basolateral medium was then collected and assessed for the presence of IL-10 (ProcartaPlex; Life Technologies Europe B.V., Thermo Fisher Scientific).

### Statistical analysis

To assess whether the effects of Biolex MB40 and Leiber Beta-S on metabolites were significantly different from the blank, two types of statistical analyses were performed. Unpaired two-sided t-tests were used to evaluate effects within any given donor, using technical replicates as input values. Paired two-sided t-tests were used to evaluate effects across the various donors, using averages of technical replicates per donor as input values.

To examine whether treatment affected alpha diversity, four common diversity measures were calculated: observed taxa, Chao1 Index, Shannon Diversity Index, and Simpson Diversity Index. Observed taxa calculates the total number of features per condition and the Chao1 Index estimates taxa richness (total number of taxa). The Shannon and Simpson Diversity Indices take both species richness (total number of species) and evenness (abundance distribution across species) into account, with the Shannon Diversity Index giving more weight to richness, and the Simpson Diversity Index giving more weight to evenness.

Beta diversity analysis was conducted by Discriminant Analysis of Principal Components (DAPC) with two discriminants (LD1 and LD2) and 80% of retained variance in the principal components using adegenet v2.1.10 [27,28].

Linear discriminant analysis Effect Size (LEfSe) was conducted on the Total-Sum Scaled (TSS) taxonomic abundances at the given taxonomic level to detect differences in community composition between conditions. Linear Discriminant Analysis (LDA) scores between treatment and individual taxon abundances were calculated using MASS v7.3.58-3. The resulting bar graph was visualized using ggplot2 v3.4.3.

To assess differences in TEER and IL-10 between the treatments and the blank control for the individual donors, a two-way ANOVA with Dunnett’s multiple comparisons test against the blank control was used. To assess differences in TEER and immune markers between the treatments and the blank control for the average of the three donors, paired, two-tailed t-test were performed, using the individual donors as replicates.

A *p* value of < 0.05 was considered statistically significant. All statistical analyses were performed using GraphPad Prism version 9.5.0 for Windows (GraphPad Software, San Diego, California, USA).

## Results

### Microbial community activity

**Fig 1:**
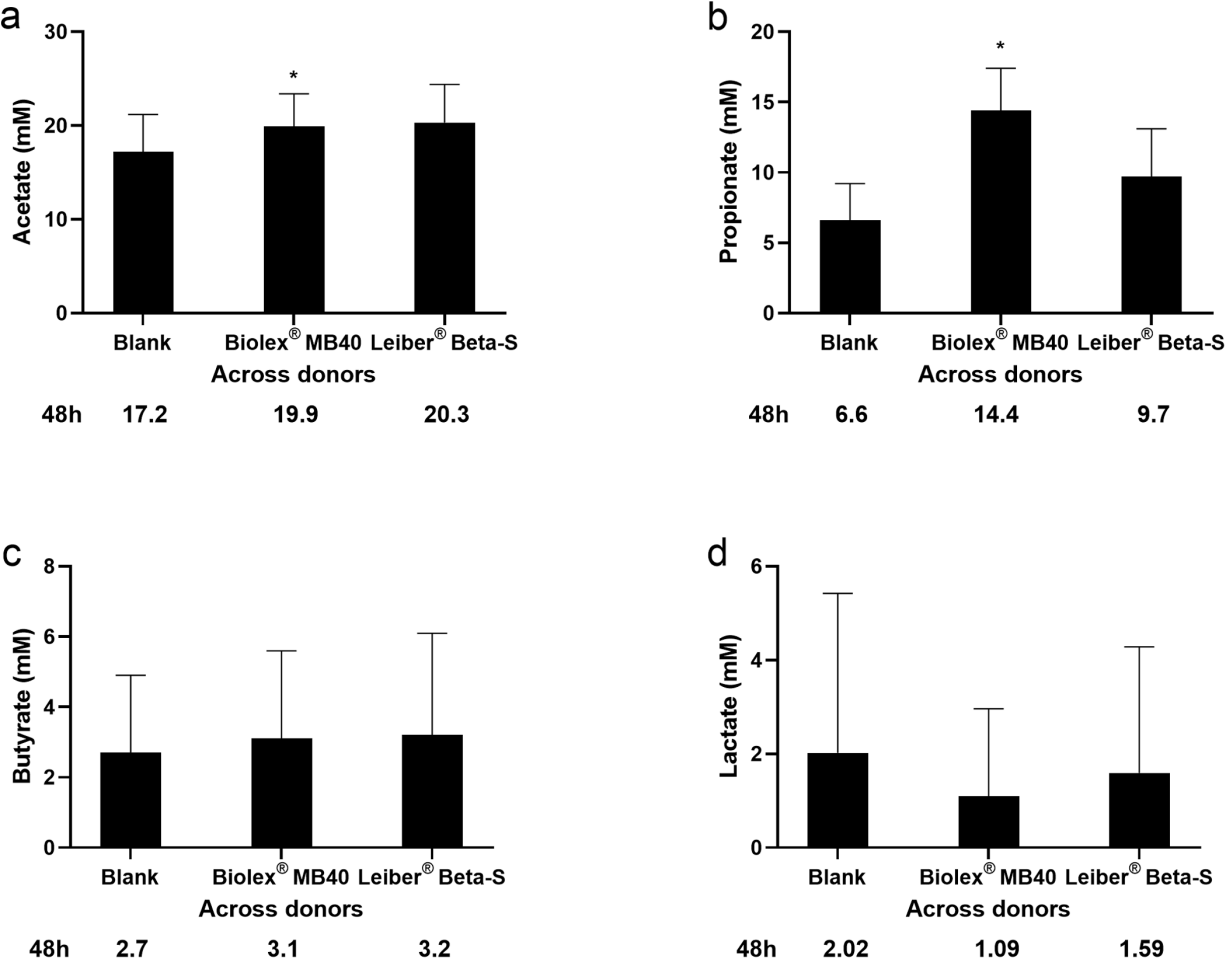
Average concentrations of short-chain fatty acids and lactic acid across all donors after 48h incubation with blank, Biolex MB40, or Leiber Beta-S in the Colon-on-a-plate system: acetate (a), propionate (b), butyrate (c), and lactate (d). Paired two-sided t-tests were used to evaluate treatment effects across all donors (treatment vs blank). *p < 0.05.

SCFA and lactate concentrations were measured at 48h. Across all donors, the acetate concentration was significantly higher in wells that received Biolex MB40 compared with blank (*p* < 0.05) (Fig 1a). Similarly, propionate concentrations were significantly higher with Biolex MB40 (*p *< 0.05) (Fig 1b). There were no significant differences in butyrate or lactate concentrations with either test product compared to blank when comparing data across all donors (Fig 1c, d).

**Fig 2:**
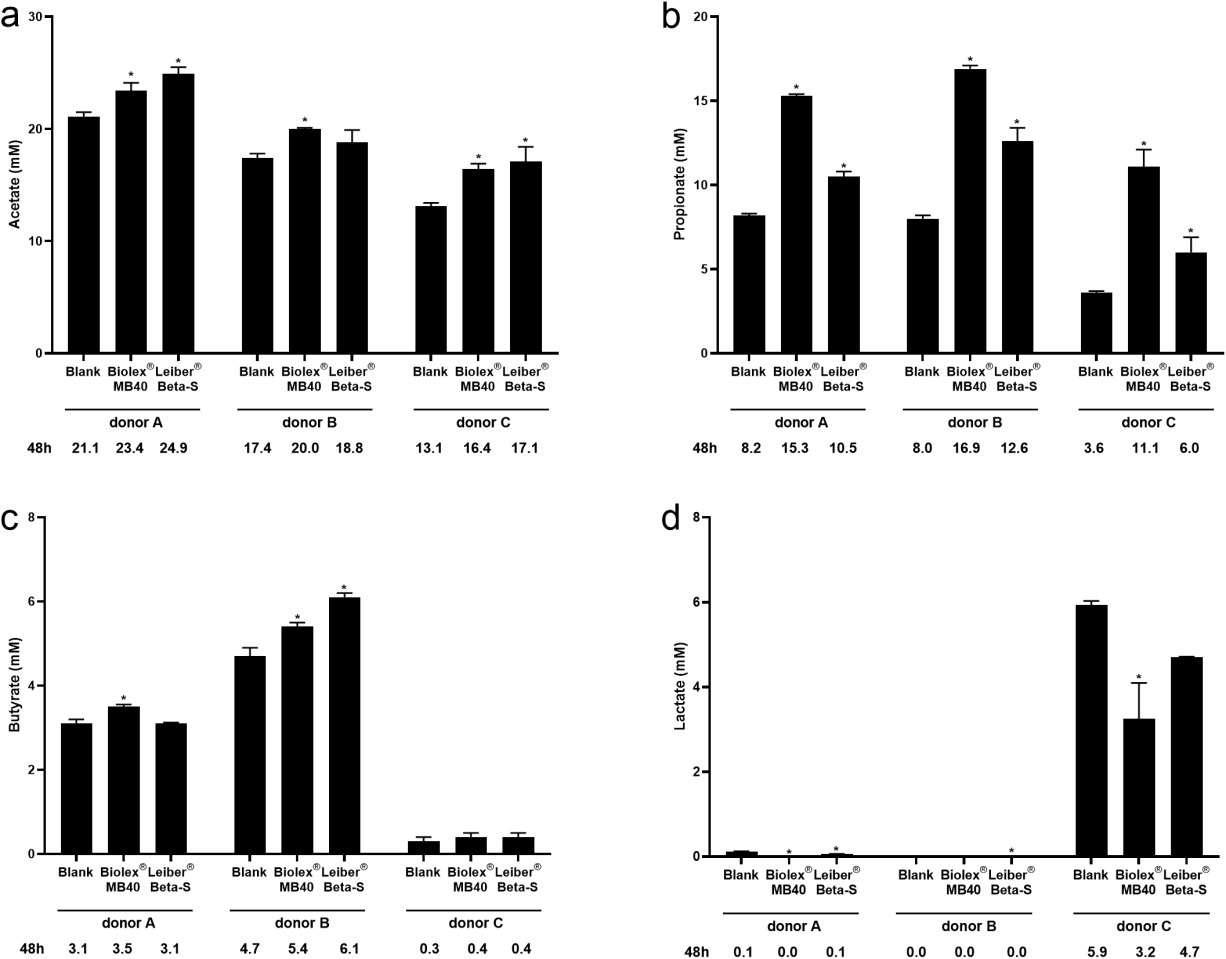
Concentrations of short-chain fatty acids and lactate for each individual donor after 48h incubation with blank, Biolex MB40, or Leiber Beta-S in the Colon-on-a-plate system: acetate (a), propionate (b), butyrate (c), and lactate (d). Unpaired two-sided t-tests were used to evaluate treatment effects within an individual donor (treatment vs blank). *p < 0.05.

When looking at SCFA and lactate concentrations for individual donors, acetate concentrations were significantly higher than blank with both Biolex MB40 and Leiber Beta-S for donors A and C, but only with Biolex MB40 for donor B (Fig 2a). Propionate concentrations were significantly higher with both Biolex MB40 and Leiber Beta-S compared with blank for all donors (all *p *< 0.05) (Fig 2b). Butyrate concentrations varied widely among the three donors. Butyrate concentrations versus blank were significantly higher with Biolex MB40 (*p *< 0.05) but not Leiber Beta-S for donor A, significantly higher with both test products for donor B (both *p *< 0.05), and differences were non-significant with either test product for donor C (Fig 2c). Notably, butyrate concentrations were virtually absent for donor C. This is also the reason that significance was not reached with the test products across all donors. Lactate concentrations were quite low for donors A and B, and relatively higher for donor C (Fig 2d). Significant differences between both Biolex MB40 and Leiber Beta-S compared with blank for donor A and between Leiber Beta-S and blank with donor B were observed; however, the concentrations were so low that the biological significance of this is unclear. With Biolex MB40, lactate concentrations were significantly lower compared with blank for donor C (*p *< 0.05), but not with Leiber Beta-S.

### Microbial community composition

**Fig 3:**
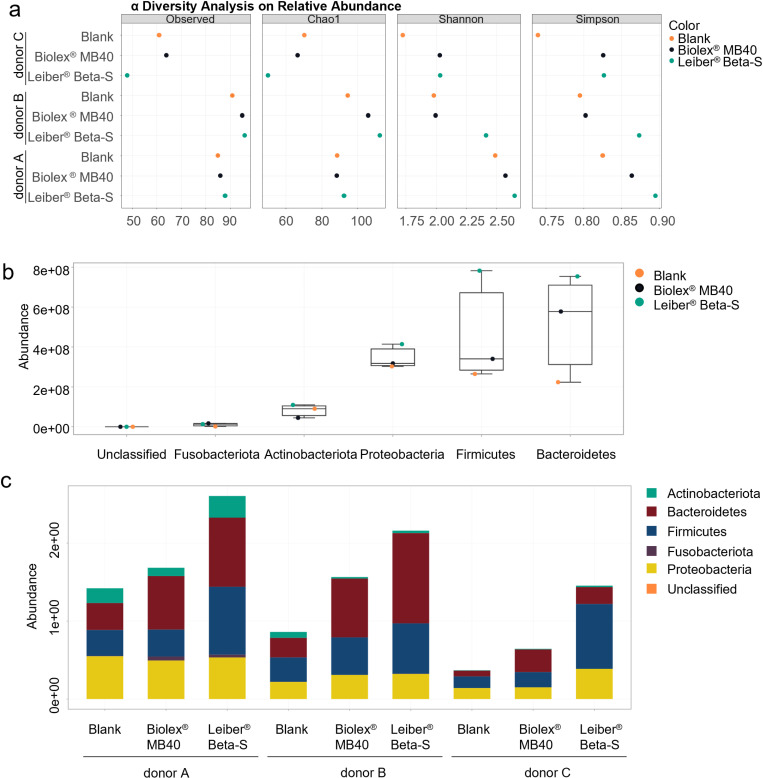
Analysis of the effects of Biolex MB40 and Leiber Beta-S 48h after the start of the Colon-on-a-plate experiment: alpha diversity analysis (observed taxa, Chao 1 diversity index, Shannon diversity index, Simpson diversity index) for each individual canine donor with inflammatory bowel disease (a), microbial community composition at the phylum level across all donors (b), and microbial community composition at the phylum level for each individual donor (c).

**Fig 4:**
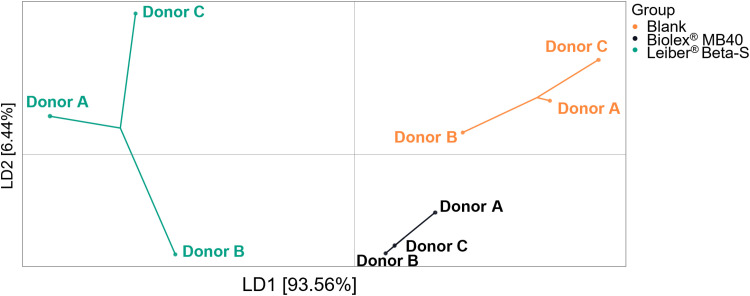
Analysis of beta-diversity in the various conditions 48h after the start of the Colon-on-a-plate experiment, using Discriminant Analysis of Principal Components (DAPC). Sequence data are transformed using principal component analysis (PCA), and subsequently clusters are identified with discriminant analysis (DA). The DA aims to maximize among‐group variation and minimize within‐group variation. In this approach, the groups (treatments) used in the DA were a priori defined.

Biolex MB40 increased the alpha diversity relative to blank in each donor and for all four indices, except for the Chao1 Index for donor A (Fig 3a). A similar effect was observed with Leiber Beta-S, showing an increased alpha diversity relative to blank in each donor and for all four indices, except for the observed index for donor C. In most cases, the increase in alpha diversity was greater with Leiber Beta-S than Biolex MB40. There was a strong increase in bacterial cell densities (abundance) with Biolex MB40 and Leiber Beta-S, with the strongest increase noted for Leiber Beta-S (Fig 3b, c). Analysis of beta-diversity was performed to assess whether either treatment induced shifts in gut microbiota composition (Fig 4). The respective DAPC plot indicates that mostly Leiber Beta-S treatment induced shifts in gut microbiota composition, as demonstrated by the shift along the LD1 axis (93.56%). The shift induced by Leiber Biolex MB40 was primarily along the LD2 axis, which is significantly less impactful (6.44%) as compared to LD1. These results indicate that Leiber Beta-S had the strongest impact on microbial community composition. The dominant bacterial phyla were Bacteroidetes, Firmicutes, Proteobacteria, Actinobacteriota, and Fusobacteriota. The phyla with the greatest increase with the test products versus blank were Bacteroidetes and Firmicutes, and these were most established for Leiber Beta-S treatment. Furthermore, at the bacterial genus level, an increase in the abundance of *Erysipelatoclostridium* was associated with Leiber Beta-S treatment, with statistical significance across donors reached. Other than this, no differences were observed at the lower taxonomic levels family or genus between treated conditions and control, likely attributed to the low statistical power associated with the low number of donors.

### Analysis of host-microbe interactions

**Fig 5:**
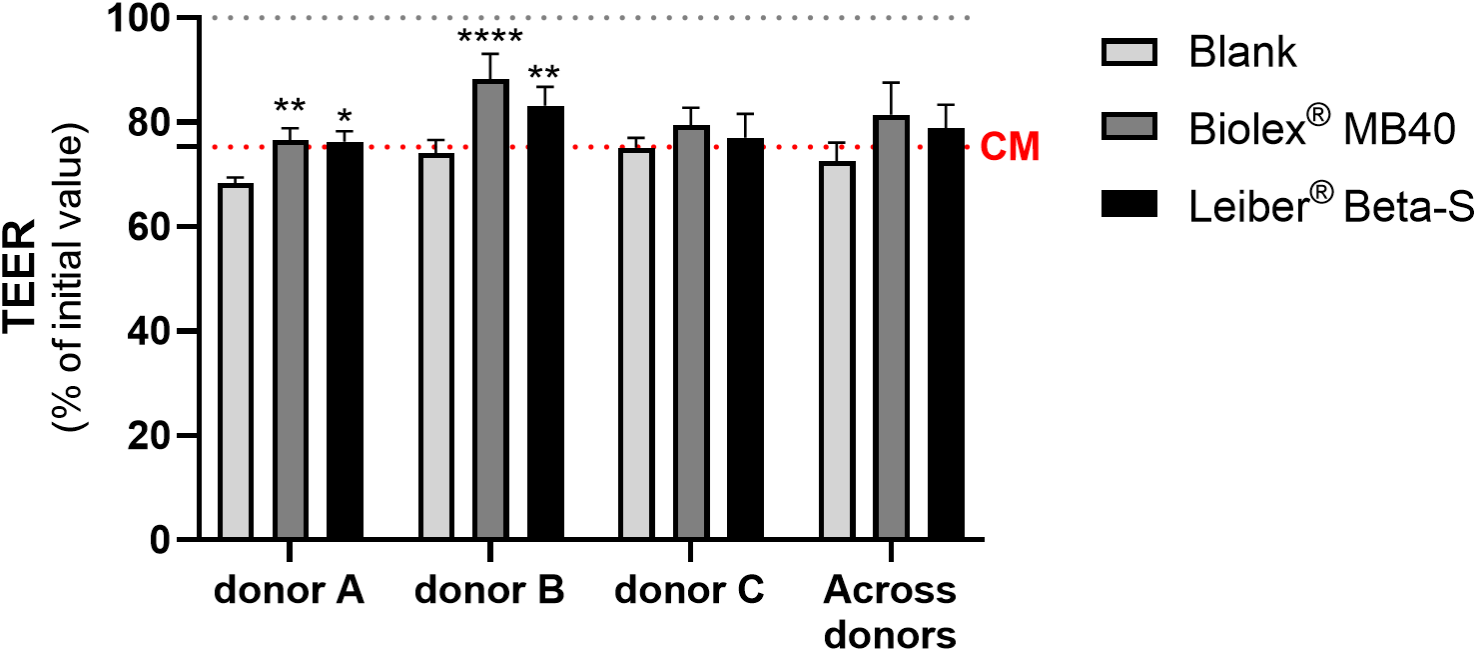
Effect of colonic suspensions on TEER of the Caco-2/THP1-Blue co-cultures. TEER was measured 24h after pre-treatment of the co-cultures with colonic suspensions and each 24h value was normalized to its corresponding 0h value and is shown as percentage of initial value. The grey dotted line represents 100% (initial value). The red dotted line corresponds to the experimental control CM (complete medium). Data are plotted as mean ± standard deviation. Each condition was tested in biological triplicate. To assess differences in TEER between Biolex MB40 or Leiber Beta-S and the blank control for the individual donors, a two-way ANOVA with Dunnett’s multiple comparisons test against the blank control was used. To assess differences in TEER between Biolex MB40 or Leiber Beta-S and the blank control for the average of the three donors, paired, two-tailed t-tests were performed, using the individual donors as replicates. *p < 0.05; **p < 0.01; ****p < 0.0001. Abbreviation: TEER, transepithelial electrical resistance.

Looking at barrier integrity, for both donors A and B, both test products had a significantly higher TEER (% of initial value) compared with blank (donor A: Biolex MB40 *p* < 0.01, Leiber Beta-S *p *< 0.05; donor B: Biolex MB40 *p* < 0.0001, Leiber Beta-S *p *< 0.01) (Fig 5). The TEER value (% of initial value) was not significantly different between test treatments and control for donor C (Fig 5). This, in turn, resulted in a lack of statistical significance between the test products and blank when the data were analyzed across all donors.

**Fig 6:**
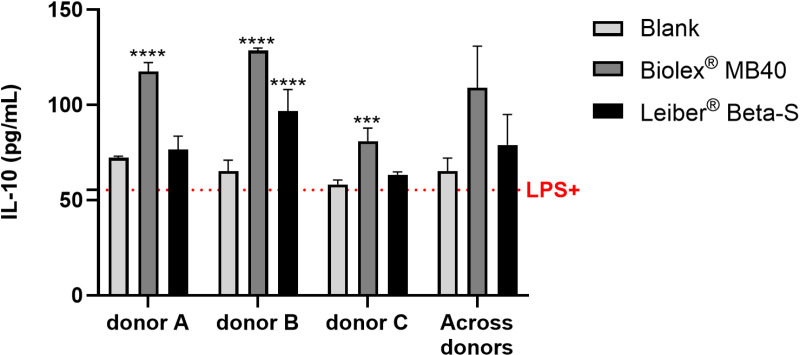
Effect of colonic suspensions on secretion of IL-10. Cytokine levels were measured 6h after LPS treatment on the basolateral side of the Caco-2/THP1-Blue co-cultures after pre-treatment of the apical side for 24h with the colonic suspensions. The red dotted line corresponds to the experimental control LPS + . Data are plotted as mean ± standard deviation. Each condition was tested in biological triplicate. To assess differences in IL-10 between Biolex MB40 or Leiber Beta-S and the blank control for the individual donors, a two-way ANOVA with Dunnett’s multiple comparisons test against the blank control was used. To assess differences in IL-10 between Biolex MB40 or Leiber Beta-S and the blank control for the average of the three donors, paired, two-tailed t-tests were performed, using the individual donors as replicates. ***p < 0.001; ****p < 0.0001. Abbreviation: IL, interleukin; LPS, lipopolysaccharide.

Colonic fermentation of Biolex MB40 increased the secretion of the anti-inflammatory cytokine IL-10 compared with blank in all donors (donor A, *p* < 0.0001; donor B, *p* < 0.0001; donor C, *p* < 0.001) (Fig 6). Colonic fermentation of Leiber Beta-S increased IL-10 secretion in all donors, which reached significance in donor B (*p* < 0.0001) (Fig 6). Due to interindividual differences, the increased secretion of IL-10 with the test products was not statistically significant when analyzed across donors.

## Discussion

Both Biolex MB40 and Leiber Beta-S were fermented by the colon microbiota of dogs with IBD. Acetate and propionate concentrations were significantly and numerically increased with Biolex MB40 and Leiber Beta-S, respectively, compared with blank. Butyrate concentrations were not significantly different from blank for either test product when looking across all donors, but they were significantly different from blank with two of the three donors when examined individually. The test products also increased biomass and alpha diversity relative to blank for all three donors, and resulted in improved intestinal barrier function (TEER) and secretion of anti-inflammatory IL-10.

A study evaluating the effects of Biolex MB40 supplementation on fecal suspensions from healthy dogs using the Simulator of the Canine Intestinal Microbial Ecosystem (SCIME) reported significant increases in acetate, propionate, and butyrate with supplementation versus control [29], which is generally in agreement with the findings of the present study. Acetate concentrations were significantly increased with Biolex MB40 supplementation relative to blank, and numerically higher with Leiber Beta-S supplementation. Large interindividual differences in effect size for acetate with Leiber Beta-S supplementation prevented significance from being reached; however, this increase was considered biologically relevant.

Biolex MB40 had a very strong propiogenic effect, simulating propionate production in each donor, with an average increase of more than twice that of blank. This strong propiogenic effect is in line with observations from the SCIME study, which reported the strongest metabolite effect of Biolex MB40 for propionate [29]. As with the acetogenic effect with Leiber Beta-S, statistical significance in propionate concentrations versus blank was not reached because of interindividual differences in effect size, but the effect was considered biologically relevant.

Regarding butyrate concentrations, minimal butyrate was detected for donor C. Supplementation with the test products in the short-term (48h) did not alter the butyrate concentration for the fecal microbiota of that donor, most likely reflecting severe dysbiosis. Butyrate concentrations were significantly increased with Biolex MB40 supplementation for both donors A and B, and with Leiber Beta-S supplementation for donor B, indicating a donor-dependent butyrogenic effect of the test products.

The low concentrations of lactate observed for donors A and B may signal a high level of cross-feeding, particularly the consumption of lactate to produce beneficial propionate and/or butyrate [30]. The high concentrations of lactate observed for donor C likely correlate with dysbiosis, indicating a lack of cross-feeding within the microbiota of this donor. A study by Minamoto et al. reported significantly lower fecal concentrations of acetate and propionate in dogs with IBD compared with healthy dogs [31]. Given that our study found an increase in SCFAs with Biolex MB40 or Leiber Beta-S supplementation, we speculate that these products could help increase SCFAs to healthier levels in dogs with IBD.

The dominant phyla in the gut microbiota of the three dogs with IBD in our study were Firmicutes, Bacteroidetes, Actinobacteria, Proteobacteria, and Fusobacteriota. We observed an increase in the abundance of each phylum with both test products; the increase in abundance was greatest for the Firmicutes and Bacteroidetes phyla, and with Leiber Beta-S supplementation. A study evaluating the effects of feed supplementation with Biolex MB40 in healthy sled dogs found that supplementation resulted in a significant increase in the abundance of Actinobacteria, but not in other phyla [32]. Using the SCIME model with healthy donors, Van den Abbeele et al. reported an enrichment of Actinobacteria in the mucosal environment and an increase in the abundance of Firmicutes in the lumen with Biolex MB40 supplementation [29]. As our study evaluated the effects of Biolex MB40 and Leiber Beta-S on the gut microbiota of dogs with IBD, we would expect some differences compared with supplementation for healthy donor microbiota. The Minamoto et al. study reported a decrease in bacterial diversity and richness in the feces of dogs with IBD relative to healthy controls, indicating that the microbiome of dogs with IBD is in dysbiosis [31]. Dogs with IBD are reported to have lower abundances of Fusobacteriota, Bacteroidetes, and Firmicutes compared with healthy dogs [4]. With both Biolex MB40 and Leiber Beta-S supplementation, alpha diversity was increased, representing an increase in bacterial diversity and richness, and an increase in the abundance of all major phyla was observed as also shown in a randomized, double blinded study in dogs with mild IBD [33]. Together, these findings confirm the prebiotic effects of Biolex MB40 and Leiber Beta-S and demonstrate that the test products may help restore the gut microbiota of dogs with IBD. Due to the low number of donors included in the study, and the strong interindividual differences typically associated with gut microbiota composition, statistical power was too low to detect consistent shifts at lower taxonomic levels (family or genus). One bacterial enrichment that was found to be consistent across donors involved *Erysipelatoclostridium* upon Leiber Beta-S treatment. The genus ferments protein to produce acetate and lactate, which could explain the increase in acetate production (and of downstream metabolite butyrate in some donors through acetate/lactate conversion) that was associated with treatment.

Increased intestinal permeability is observed in both humans with IBD and animal models of IBD, thus, it is considered an important component of the disease [34]. We found that colonic suspensions from donors A and B supplemented with Biolex MB40 and Leiber Beta-S significantly increased the TEER value of Caco-2 cells relative to blank following the triggering of inflammation by co-culturing with activated macrophages. This indicates a reduction in intestinal permeability and protection from inflammation-induced barrier disruption. No increase in TEER was observed for donor C. These findings are supported by an *in vivo* study in healthy sled dogs that either did or did not receive Biolex MB40 supplement for 10 weeks [35]. That study found that markers of intestinal permeability were reduced in dogs following supplementation, but that no change was observed for dogs that did not receive the supplement. Together, these data suggest that Biolex MB40 or Leiber Beta-S supplementation may improve gut barrier integrity in dogs with IBD.

Colonic fermentation of Biolex MB40 had anti-inflammatory properties as demonstrated by the significant increase in the production of the anti-inflammatory cytokine IL-10 in the Caco-2/THP1 co-culture model with all three donors; fermentation of Leiber Beta-S resulted in increased IL-10 production with all donors, which only reached significance for donor B. This is in agreement with a study evaluating β-1,3–1,6-D-glucan supplementation in dogs with IBD that reported an increase in serum IL-10 concentrations [20].

Metabolites of the test products are likely to be responsible, at least in part, for the effect of colonic supernatants on the intestinal barrier and IL-10 production in the *in vitro* Caco-2/THP1 co-culture model. SCFAs have protective effects on inflammatory intestinal barrier disruption and modulate cytokine production by epithelial cells. Butyrate is a main energy source for colonocytes and has a role in modulating signaling pathways and transcription factors with an end result of increased epithelial barrier function and decreased inflammation [36]. Butyrate has been shown to increase anti-inflammatory mediators, such as IL-10, following NFκB activation, as would occur with LPS stimulation [36]. The increase in butyrate can be linked to the increase in relative abundance in Firmicutes with the test products, as most of the butyrate-producing bacteria found in the gut are members of the Firmicutes phylum [37]. These microbes utilize both acetate and lactate to produce butyrate via cross-feeding [30]. Propionate has also been shown to improve intestinal permeability and decrease LPS infiltration into the blood [38]. Thus, the strong propiogenic effect observed with test product supplementation also likely played a role in the improvement of intestinal permeability and increase in IL-10 production. The increase in propionate was likely due to the increased relative abundance of the phylum Bacteroidetes, which contains many propionate producers [37].

This study had a few limitations. First, given that it was conducted using an *in vitro* model, the findings might not be directly translatable to the *in vivo* condition. Second, the number of donors included in the study was limited, and could benefit from including a larger number, the more considering the heterogeneity that is expected for this type of disease. Regardless, a rather homogeneous response across donors in terms of production of acetate, propionate, and IL-10 was observed. Likewise the impact on barrier integrity was rather homogeneous across dogs. The study therefore provides first mechanistic insights in how the tested treatments benefit dogs having been diagnosed with IBD. It is expected that the biggest limitation of the low number of donors results from the low statistical power to detect consistent shifts in microbial community composition. However, since it is the actual metabolites that contribute to gut-health, metabolic shifts are generally considered most important, while shifts in community composition remain mostly descriptive. Regardless, it is advised that treatment effects are validated *in vivo*, and this on a significantly higher number of donors.

## Conclusion

Overall, this study found that Biolex MB40 or Leiber Beta-S were fermented by the gut microbiota of dogs with IBD, and that supplementation had several *in vitro* effects which are considered beneficial. Fermentation of the two test products resulted in increases in health-promoting SCFAs, increases in biomass and bacterial diversity and richness, provided protection against inflammation-induced gut barrier permeability, and stimulated IL-10 production. Together, these findings confirm a prebiotic effect for both test products and indicate that Biolex MB40 or Leiber Beta-S supplementation may provide health benefits for dogs with IBD by modulating several aspects of the gut microbiome.

## Supporting information

S1 File Supplementary data. The S1 File contains the raw data used to generate the figures in the manuscript. These data include the replicate values (n = 3) of all test conditions (3 donors, blank, 2 products) for acetate, propionate, butyrate, lactate, TEER (presented as % of initial value) and IL-10. Values in red indicate outliers, excluded from the calculations. In addition, the file contains a table with the raw data of the 16S sequencing showing the abundance of all represented OTUs as absolute and relative values and the bacterial cell counts obtained by flow cytometry.
(XLSX)

S1 Table Clinical data and histopathological findings of three canine fecal donors. (DOCX)
