# Supplementary material for: In vitro fermentation of yeast cell walls (mannan-oligosaccharide) and purified β-glucans modulates the colonic microbiota of dogs with inflammatory bowel disease and demonstrates protective effects on barrier integrity and anti-inflammatory properties
Source: PLoS One. 2025 May 13;20(5):e0322877. doi: 10.1371/journal.pone.0322877 (PMC12074461; doi:10.1371/journal.pone.0322877)
Supplement: S1 Table — (DOCX) [file pone.0322877.s002.docx]

**Supplementary table 1. Clinical data and histopathological findings of three canine fecal donors.**

|  | Signalment | Clinical signs | Food trial completed | Endoparasites excluded | Current medical treatment | Histopathological findings |
| --- | --- | --- | --- | --- | --- | --- |
| 1 | 7-years old female spayed Boxer | Chronic (3 months) vomiting, small intestinal diarrhea, nausea, weight loss, lethargy | Yes (Purina HA) | Yes | Bedinvetmab 15mg SC q 4 weeks | Stomach: chronic, mild to moderate lymphoplasmacytic and neutrophilic gastritis with multifocal mild lymphoid follicular hyperplasia  Duodenum: chronic, mild lymphoplasmacytic, neutrophilic and less pronounced eosinophilic enteritis with mild villous blunting and mild proprial fibrosis.  The cause of the inflammation is not  apparent at either site sampled: significant bacteria, endoparasites, foreign material and neoplasia are not observed. |
| 2 | 2-years old female spayed Crossbreed | Chronic (6 months) vomiting, intermittent melena | Yes (homemade diet combined with Trovet hypoallergenic (Venison, VPD) | Yes | Omeprazole 1mg/kg q12hours  Metoclopramide 0.3 mg/kg q 12hours  Maropitant 2 mg/kg q24hours | Stomach: chronic, mild to moderate lymphoplasmacytic and neutrophilic gastritis with mild pyloric mucosal hyperplasia (focally extensive).  Duodenum: chronic, mild lymphoplasmacytic and neutrophilic enteritis.  The cause of the inflammation is not  apparent at either site sampled: significant bacteria, endoparasites, foreign material and neoplasia are not observed. |
| 3 | 3-years old female spayed Cavalier King Charles Spaniel | Chronic (9 months) vomiting, nausea, hyporexia, small intestinal diarrhea, one episode of melena | Yes (Hills z/d) | Yes | Omeprazole 1 mg/kg q24hours  Maropitant 2 mg/kg q24hours | Stomach: chronic, mild lymphoplasmacytic, neutrophilic and eosinophilic gastritis.  Duodenum: chronic, multifocal, mild lymphoplasmacytic, neutrophilic and eosinophilic enteritis with mild multifocal villous blunting.  The cause of the inflammation is not  apparent at either site sampled: significant bacteria, endoparasites, foreign material and neoplasia are not observed. |

q, every; SC, subcutaneous
